# 4365 Family-Based Study of Sleep in Autism Spectrum Disorder without Intellectual Disability

**DOI:** 10.1017/cts.2020.236

**Published:** 2020-07-29

**Authors:** Stacey Elkhatib Smidt, Arpita Ghorai, Brielle Gehringer, Holly Dow, Zoe Smernoff, Sara Taylor, Jing Zhang, Daniel Rader, Laura Almasy, Edward Brodkin, Maja Bucan

**Affiliations:** 1 University of Pennsylvania School of Medicine

## Abstract

OBJECTIVES/GOALS: Autism spectrum disorder (ASD) is characterized by difficulties in communication and social interaction as well as restricted and repetitive behaviors. Sleep problems are a common concern in children with ASD that can persist into adulthood. This study aims to further explore sleep in ASD without intellectual disability (ASD w/o ID). METHODS/STUDY POPULATION: We recruited individuals of both sexes with ASD w/o ID (probands) and relatives as part of the Autism Spectrum Program of Excellence (ASPE) at the University of Pennsylvania. Actimetry data were collected via a wrist-worn tri-axial accelerometer for 21 days. Data from 212 participants were considered. We analyzed sleep data using the algorithms GGIR, ChronoSapiens, and PennZzz. The sleep traits of proband and sibling pairs were compared using paired t-test or Wilcoxon signed-rank test. We used the Social Responsiveness Scale, Second Edition (SRS-2) to assess social impairment and restricted/repetitive traits. We compared SRS-2 scores to sleep traits using partial Spearman or Pearson correlations adjusting for age (171 participants). RESULTS/ANTICIPATED RESULTS: Probands demonstrated later sleep onset (p = 0.03), decreased M10 average (10-hour period of highest activity/day; p = 0.006), decreased relative amplitude (measure of rest-activity rhythm; p <0.001), and decreased total daytime activity (p = 0.005) compared to siblings. Regarding social function and restricted/repetitive traits, adult males showed an inverse correlation between SRS-2 total score and sleep efficiency (r = −0.2, p = 0.04) and a positive correlation between SRS-2 total score and intradaily variability (r = 0.3, p = 0.02). Adult females showed an inverse correlation between SRS-2 total score and M10 average (r = −0.3, p = 0.02) and between SRS-2 total score and relative amplitude (self-report r = −0.4, p = 0.001; informant r = −0.3, p = 0.005). DISCUSSION/SIGNIFICANCE OF IMPACT: This study focuses on the analysis of sleep traits in ASD including the relationship between social function and sleep. Thus far, the most robust findings are decreased daytime activity and relative amplitude in individuals with ASD w/o ID compared to siblings. We have also shown that ASD social impairment may be related to sleep dysfunction.

